# Train up a Child

**Published:** 1872-01

**Authors:** Ben. H. Pratt


### THE BISTOURY.

VOL. VII. ELMIRA, N. Y., JANUARY, 1872. No. 4.

### "TRAIN UP A CHILD."

BEN. H. PRATT, A. M.

If men and women could retain their memories of childhood—did not cease to remember that they were once boys and girls—could identify themselves more thoroughly with the thoughts, feelings, emotions and passions of their own and other's children—there would be fewer homilies written, and less talking done, upon the raising of children, family government, early training, and the like. Solomon was reputed a very wise man, and edited some very sage saws on the theory of training children. Many still accept his generalities as all sufficient, and we observe the excellent effects thereof in the remarkably proper specimens of manhood and womanhood, which emanate from the homes of Ministers and Deacons. "Train up a child in the way he should go," is Bible law, and I very much dislike to gainsay its teachings; and yet no more pernicious practice obtains than the close observance of this same bit of Scripture. The misinterpretation of this text, and not the spirit and intention with which Solomon indited it, does the harm. An unwarrantable elipsis is supplied, making the text read: "Train up a child in the way he should go after he has arrived at maturity"—that is, make a man out of a boy—compel a five-year old head to carry a twenty-year old brain—expect a colt to do a horse's work.

I don't mean to theologize, nor commentate, nor pulpitize; in fact, I am sometimes inclined to think that good, square Common Sense is too often ignored in those operations. This important ingredient in the manufacture of opinion, applied to Solomon's ipse dixit, makes it mean "Train up a child in the way a *child* should go"—and here lies the secret of efficient *training*.

To put yourself in the child's place; think the child's thoughts and feel his own little joys and sorrows, is as difficult as it is necessary; especially so for those who have interested themselves but little in children for a period of years. Yet, if you only know it, such association is one of the preventives of premature age, and keeps the spring of youth constantly a-bubble in your heart. Stern and uncompromising rules for the guidance of a child, never made a healthy mind nor evoked a buoyant spirit.—Love is perhaps a more pernicious ruler than fear, in home government, if unaccompanied by an adaptation of the governing to the governed mind. Love alone gives loose reigns to every whim and fancy of childhood, and makes the child the master and the parent the servant.—Fear alone, makes a slave of the child and a tyrant of the parent. Parental love, tempered with a memory of what our own childhood craved, what it delighted in and what it sighed for; a hearty co-operation with, and tender regard for, all the little ambitions and aspirations which enter into every-child-life—in a word, a true intimacy and free interchange of sentiment between parent and child—these make our great and good, and natural men and women. A life cannot be suddenly and peremptorily bent without being broken—it *can* be gently drawn and coaxingly made to grow into a sublime rectitude.

Not having space to enter into minute detail upon this subject, I will only enumerate a few of the leading points upon which I regard many parents at fault.

Fun is natural to the young. Fun is *not* natural to maturer years, and the older we grow the less we appreciate fun.

Here begins a difficulty at once. The parent who may not care for any fun, cannot sympathise with the child that cares for little else but fun. In the gleeful pursuits of its natural stimulus, suddenly falls the fiat of parental authority, and checks this element of childhood's joy, and the flame of its happiness by oft-repeated drenchings, become well nigh quenched, and that most pitiful of all sights is fo rced uponus a serious child. Excitements are as needful to children as food. Not excitement such as older people crave, but that constant charge of occupation which is sought for, the house over.—The little one roams from spot to spot, and room to room, and floor to floor, dilligently conjuring up a new play and new scenes, much to the annoyance of the unsympathising mother, or the fear of an over-prudent father.

Growing older, games of simple sort awake the child to a new world, and its inborn love for these grows with its growth and strengthens with its strength. In this, the anxious parent is too apt to see an imaginary ill in the future. Games begin to be frowned upon, and by and by excluded from the home circle, lest they lead to cards and dice, the horror of many otherwise sensible folk. Romping games for girls and boys begin to approach that terror for some people, dancing—and all such are tabooed.—Tableaux and charades are forbidden, lest they arouse a taste for the drama, and all entertainments of a public character are avoided for fear of an ill they know not of. That people should live to be fathers and mothers and not acquire a sufficient knowledge of human nature to know that no fruit is so sweet as the forbidden—and naught so charms as that which is constantly denied, is one of the unsolved mysteries. Childhood is a sly minx, and will effect by stratagem what it cannot by fair means. Thus little Tom and Susy will steal away to the house of some less scrupulous neighbor, to learn the very things they cannot learn at home, and learn besides what is far worse, how to practice deceit, and pull the wool over parental eyes.

Better far it is to allow our children to do at home, even objectionable things, than indirectly teach them to do what is incontrovertibly wrong. Card-playing has been discussed pro and con—it may be an evil—it may not. I will not enter upon the question. But I do know that I have never yet heard the straightest, strictest, most prejudiced opponent to the practice, even admit that there was anything wrong in the playing of cards, per se—the associations and oft times accompaniments are the objectionable features.—Shorn of evil associations, then, card playing becomes simply an innocent and agreeable pastime—and the keen edge of curiosity is taken from all desire to visit improper places for this amusement. Dancing at home, at seasonable hours, with members of your own family and other proper persons, is absolutely innocent.—Indeed, there is no other amusement that so completely combines exercise for the body, recreation for the mind, and affords grace of motion and easy bearing in society.

A moderate and prudent attendance at places of public amusement of an elevated character and good moral tone, satisfies that craving in childhood for exhibitions of this sort, and removes in most cases any desire to attend the countless disgraceful *shows* that infest every community.

The parent grown, however, forgets what he used to think in matters of this kind. He fails to remember and profit by his own youthful reasoning, which ran something in this wise :—"I don't see why father won't let me have any fun at home. He sits all the evening and reads that endless newspaper, and looks cross if I speak, or drop a plaything. And when any one comes to see me, I can't entertain them, for it annoys him so. I can't go to parties, 'cause I can't dance, and nobody likes my parties, 'cause father's so strict. And how I would just like to go to the theatre once to see those splendid things the boys at school tell about, and wouldn't I give all my savings to go to dancing school. Well, I know what I'll do when I get to be a man.—I'll go every where and do everything any body else does, and make up for all these stupid hours at home." This is a synopsis of the mental operations of every child too strictly governed at home, and it don't need much argument to prove that when the opportunity arrives, boys do indulge in the denied fascinations immoderately, to their harm sometimes—sometimes to their ruin. Far be it from me to put a viper into the bosom of any family, but I sincerely believe, both from observation and experience, that a vast amount of harm is done in the family by what is termed careful training. I would not advise what I do not purpose doing myself, and what I have seen done with the happiest effects. Checkers, chess, back-gammon, cards and even billiards shall always have a place in my family so long as my means will admit of it. Music and dancing shall never be frowned upon, but encouraged, for I believe in them, not only as elegant, but wholesome educators. I would not urge attendance upon miscellaneous public entertainments, but judiciously select such as I believed to be of an elevated and elevating character,and be my own family escort. Even improper places, openly visited for the purpose of pointing a moral, are far better lessons than are sure to be learned some day furtively.

Again, I would urge a greater familiarity between parents and children. Letting ourselves down to the comprehension of our children is sure to produce an effort on the children's part to reach upward toward us. I fear for the child that fears a parent. I pity the child that don't like to worry father nor mother with a question. The want of intimacy which such feelings evince, is a sad commentary upon the home government. Happy the parent that can enter into and enjoy his children's pleasures, and sympathize with them in their sorrows—who can let himself down to them, hoping thereby to bring them up to him. This need not necessarily make pert, or forward, or bold children, but by respecting the opinions and ingenuous remarks they make, encourage an independence of thought and manner that cannot but prove both a pleasure to the parent and a profit to the child.

In fine, let us not be too "wise in our own generation," and by too careful and over prudent rearing of our children, entail upon them the very ills we seek to avoid.

